# S80. Prolongation of T cell response by OX40 co-signalling CARS

**DOI:** 10.1186/2051-1426-2-S2-I18

**Published:** 2014-03-12

**Authors:** H Abken

**Affiliations:** 1 Klinik I fur Innere Medizin, Koln, Germany

## 

Adoptive therapy of cancer with genetically redirected T cells showed spectacular efficacy in recent trials. A body of pre-clinical and clinical data indicate that young effector and central memory T cells perform superior in a primary anti-tumor response; repetitive antigen engagement, however, drives T cell maturation to terminally differentiated cells associated with the loss of CCR7 which enables T cells to persist in peripheral tissues. Chimeric antigen receptor (CAR) engineered CCR7^-^ T cells more efficiently accumulated at the tumor site, secreted more IFN-g, expressed higher amounts of cytotoxic molecules and showed superior tumor cell lysis compared to the younger CCR7^+^ cells. CCR7^-^ T cells, however, were more prone to spontaneous and activation induced cell death which could be counteracted by simultaneous CD28 and OX40 (CD134) costimulation. Consequently, the combined CD28-z-OX40 signaling CAR rescued CCR7^-^ T cells from apoptosis which then produced more efficient anti-tumor efficacy than CCR7^+^ T cells redirected by the same CAR. In contrast, cytokine induced killer (CIK) cells, predominantly consisting of terminally differentiated CD8^+^CD56^+^ cells, accelerated terminal maturation of CD56^+^ CIK cells producing high frequencies in activation induced cell death (AICD) and reduced anti-tumor efficiency when stimulated by the CD28-z-OX40 CAR compared to the CD28-z CAR. Translated into therapeutic strategies, T cell therapy will benefit from combined CD28-z-OX40 stimulation in the long-term by rescuing continuously generated CCR7^-^ T cells for an anti-tumor attack. CAR redirected CIK cells benefit from CD28 co-stimulation; "super-costimulation" by the CD28-z-OX40 CAR, however, performed less in anti-tumor efficacy due to increased AICD.

